# Structural switches: intracellular G-quadruplexes modulate DNA repair and genome maintenance in *Deinococcus radiodurans*

**DOI:** 10.1128/mbio.01639-25

**Published:** 2025-09-29

**Authors:** Himani Tewari, Shruti Mishra, Swathi Kota

**Affiliations:** 1 Molecular Biology Division, Bhabha Atomic Research Centre, Mumbai, India; 2 Homi Bhabha National Institute, Mumbai, India; Vanderbilt University School of Medicine, Nashville, Tennessee, USA

**Keywords:** *Deinococcus radiodurans*, guanine quadruplexes, DNA repair, genome maintenance, RecQ helicase

## Abstract

Guanine quadruplex (G4) structures are secondary structures formed in nucleic acids containing guanine-rich stretches. The regulatory role of G4s in various cellular processes has been studied in both eukaryotes and prokaryotes. *Deinococcus radiodurans*, an extremophile, shows increased sensitivity to ionizing radiation in the presence of G4-binding ligands during the post-irradiation recovery (PIR) period. Here, for the first time, a detailed study is reported on the intracellular formation and resolution of G4 structures in this organism. The potential G-quadruplex-forming sequences, positioned at different locations within the genes, folded into different topologies *in vitro*. The divalent cation Mn²^+^, which plays a crucial role in radioresistance, destabilized G4 structures, whereas Mg²^+^ supported the G4 formation. Depending on their location at the 5′ or 3′ end of the gene, the native G4 motifs differentially regulated the endogenous gene expression under both normal and PIR conditions. Thioflavin T and anti-G-quadruplex antibodies detected the *in vivo* formation and dynamics of G4s in response to various DNA-damaging agents, with the highest levels observed following gamma radiation treatment. The presence of G4-binding drugs during the PIR arrested G4 structure dynamics, increased the cellular levels of 8-oxo guanine, and delayed the DNA repair process. Furthermore, the absence of RecQ resulted in the accumulation of more G4 structures within the cells, leading to genome instability. These results indicate that in *D. radiodurans,* the *in vivo* formation of G4 structures varies significantly under normal and cellular stress conditions, depending on the levels of Mn²^+^ and G4 resolving proteins.

**IMPORTANCE:**

Guanine quadruplexes (G4s) can act as both activators and inhibitors of various cellular functions, depending on their genomic position. While G4 functions are increasingly understood in eukaryotes, their specific roles in bacteria, especially *in vivo*, are still largely unexplored. *Deinococcus radiodurans* harbors a GC-rich genome with numerous potential G-quadruplex-forming sequences. In this study, the first direct evidence of G4 formation *in vivo* and its dynamic nature during the DNA damage response to gamma radiation is shown in a bacterium. The significance of G4 structural dynamics in modulating endogenous gene expression, DNA repair, and genome maintenance processes is demonstrated. These findings provide a novel insight into G4-mediated regulatory mechanisms in prokaryotes and expand our understanding of G4’s role as a dynamic structural switch.

## INTRODUCTION

Guanine quadruplex (G4) structures are non-canonical secondary structures formed in DNA and RNA sequences rich in guanine. Four guanine bases pair through Hoogsteen hydrogen bonding to form a G-quartet, which, upon stacking on each other, forms G-quadruplex structures. Monovalent cations, such as Na^+^ and K^+^, play a significant role in the folding and stability of these secondary structures. G4 structures can be formed either from a single strand (intra-molecular) or from two or more strands (intermolecular) (1). The presence of intramolecular G4 structures at gene promoters and telomeres, along with their regulatory roles in gene expression and genome stability, has been extensively studied in the recent past (2, 3). Similarly, the significant contributions of intermolecular G4 structures in chromatin looping and the maintenance of long-range DNA interactions for performing various biological functions have emerged recently (4). Nevertheless, whether inter- or intra-molecular, G4 structures are now known to regulate important cellular processes in organisms, making them potential targets for various pathological conditions, including cancer (5, 6).

In bacteria, the role of G4s was first discovered in pilin antigenic variation in the human pathogen *Neisseria gonorrhoeae,* which leads to evasion of the immune system (7). Studies in other pathogenic bacteria, such as *Streptococcus pneumoniae* and *Mycobacterium tuberculosis*, have shown that G4 motifs are associated with the transcription of genes necessary for virulence (8). In *Escherichia coli,* potential G-quadruplex-forming sequences (PQS) were found to be overrepresented in the regulatory regions of the genome, including near transcription factor-binding sites (9). It was later observed that the G4-forming consensus sequences (G_3_N_1-7_G_3_N_1-7_G_3_N_1-7_G_3_) are often flanked by C-rich regions, forming G4 and hairpin-like regulatory switches in the genome (10). Recent studies have revealed the prevalence of G4-forming sequences within the *Helicobacter pylori* genome, often located in regulatory regions of genes essential for bacterial survival and virulence (11). Furthermore, it has been demonstrated that several bacterial helicases, such as RecQ, Rep, UvrD, and other proteins, including RecA, MutS, topoisomerases IB, play crucial roles in the resolution, interaction, or formation of G4 structures (8, 12–15). While the majority of investigations in bacteria support the significance of G4 structures in cellular processes, they still lack comprehensive evidence for the *in vivo* formation, as shown in higher organisms.

*Deinococcus radiodurans* is a gram-positive bacterium known for its remarkable resistance to many abiotic stresses, including high doses of ionizing radiation. This resistance is attributed to the presence of efficient DNA damage repair pathways and strong anti-oxidant mechanisms (16, 17). *D. radiodurans* harbors a multipartite genome consisting of two chromosomes and two plasmids with high GC content (18). Numerous PQS have been identified in this bacterium’s genome. Earlier, it has been shown that the presence of G4 stabilizing ligands like N-methyl mesoporphyrin IX (NMM) during post-irradiation recovery (PIR) makes this bacterium radiosensitive (19). Bioinformatics analyses further identified PQS in the upstream regions of DNA repair genes, such as *recA*, *recQ,* and *mutL,* which could form stable G4 structures *in vitro* (20). High-throughput transcriptome analysis of *D. radiodurans* cells in response to the G4-stabilizing ligand NMM and gamma radiation has also shown the role of PQS in regulating DNA damage-responsive gene expression (21). The extended synthesis-dependent strand annealing pathway (ESDSA) is the main DNA damage repair process that operates when the bacterium is subjected to high doses of gamma radiation (22). Single-stranded DNA (ssDNA) synthesized during the period of the ESDSA process is prone to G4 structure formation. However, how these secondary structures hinder or promote various cellular processes during the PIR period and affect the outcome of the ionizing radiation resistance phenotype remains unclear.

The present study has investigated the *in vivo* dynamics of G4s and their effects on cellular functions that contribute to ionizing radiation resistance in *D. radiodurans*. The PQS present at different positions within the gene (5′, 3′, or intergenic regions) folded into different topologies in the presence of various monovalent cations. The presence of divalent cation Mg^2+^ facilitated the *in vitro* formation of G4s, whereas G4s were not formed in the presence of Mn^2+^. The native PQS at 5′ or 3′ gene location differentially regulated transcription under gamma radiation-treated and -untreated conditions. Besides gamma radiation resistance, the mitomycin C resistance of *D. radiodurans* was also compromised when cells were treated with G4-binding ligands. Thioflavin T (ThT), a fluorescent dye, as well as antibodies that bind specifically to G4s, detected *in vivo* formation of G4 structures and their dynamics during the PIR. Further studies showed that the arrest of G4 structure dynamics slows the DNA damage repair process, and the absence of G4 helicase RecQ leads to morphological changes in this bacterium. All these results suggest that *in vivo* G4 structure formation in *D. radiodurans* is dependent on Mn^2+^ concentration as well as on proteins like RecQ, with G4s playing a crucial regulatory role in DNA damage repair and genome maintenance during gamma radiation stress.

## MATERIALS AND METHODS

### Bacterial strains, plasmids, and materials

*D. radiodurans* R1 (ATCC 13939, a gift from Ortner [Germany]) (23) was grown and maintained in TGY medium (Bacto Tryptone [0.5%], glucose [0.1%], and Bacto yeast extract [0.3%]) at 32^°^C. The bacterial strains used in this study are listed in Table S1 at https://barc.gov.in/publications/mBio01639-25R1/index.html. Anti-DNA G4 antibody clone 1H6 (MABE1126), anti-bromodeoxyuridine (BrdU) antibody (B8434), and anti-8-Oxoguanine antibody (MAB3560) were obtained from Sigma-Aldrich, St. Louis, USA. 5,10,15,20-tetrakis-(*N*-methyl-4-pyridyl) porphyrin (TMPyP4) and ThT dye 2-(4-(Dimethylamino) phenyl)−3,6-dimethylbenzo[d]thiazol-3-ium chloride were procured from Sigma-Aldrich. NMM was obtained from Frontier Scientific, Utah, USA, and all the chemicals and enzymes for molecular biology were procured from Merck Inc. and New England Biolabs. The molecular biology techniques used in this study were as described earlier (24).

### Identification of potential DNA G4-forming sequences

The PQS present at various locations within the coding region of the genes such as, at the 5′ end (downstream of the transcription start site) and the 3′ end (upstream to the stop codon), and in the intergenic regions on the genome of *D. radiodurans* were identified using Quadruplex forming G-Rich Sequences (QGRS; http://bioinformatics.ramapo.edu/QGRS/index.php) and G4 hunter (http://bioinformatics.ibp.cz/) software. For QGRS, the parameters used were (G3N _(0–11)_ G3N _(0–11)_ G3N _(0–11)_), maximum length of 45, minimum G-group 3, and loop size of 0–11. For the G4 hunter, the threshold value was 1.7, and the window size was 25. The FASTA sequence of chromosomes I and II, along with megaplasmid and small plasmid, was retrieved from the National Center for Biotechnology Information (NCBI) and analyzed using both QGRS and G4 hunter. The results obtained from the QGRS analysis, along with their genomic positions, were named and tabulated. The genes that contain the PQS within ~200 bp of the 5′ or 3′ end of the gene were selected. Some of the PQS were synthesized and studied for the formation of G4 structures as described earlier (20). The sequences of selected PQS are listed in Table 1.

**TABLE 1:** PQS forming structural motifs in the genome of *D. radiodurans* used in this study

Gene name or ORFs	Gene position	G4 motif sequence (5'−3')	G4 motif starting position	G4 motif location within the gene
DNA processing protein A, *dprA* (DR_0120)	118232–119344	GGGCGGCAGCAGGGGAGGGAAGGG	119317	5' end (template strand +27)
Partition protein A*, parA* (DR_1685)	1705511–1706122	GGGCGGGTGGGCAGTTCGCTGCGCTGGG	1705631	5' end (non-template strand +120)
DNA repair protein, *recN* (DR_1477)	1490721–1492415	GGGCTGCTGTTGGGCGGGCGGG	1490943	5' end (non-template strand +222)
NAD(P)/FAD-dependent oxidoreductase*, nfdo* (DR_A0328)	353101–354243	GGGCGGCGGGCCGGTGGGGACATTTCTGGG	353121	5' end (non-template strand +20)
Tyrosine-type recombinase/integrase*, ssi* (DR_0513)	517462–518796	GGGGCCGGGGCCGCCGCTGGGGTCAGCGGG	517598	3' end (template strand +136)
DNA damage response D, *ddrD* (DR_0326)	319806–320402	GGGCGTTCGGGGCCAGCCGGGTCGGTGGG	319996	3' end (template strand +190)
MBL fold metallo-hydrolase*, mbl* (DR_A0069)	75567	GGGAAGGGGCAGGGGGAAGGCGGG	75538	3' end (non-template strand +6)
Guanine deaminase*, guaD* (DR_A0180)	184861–186180	GGGTGTGGGTCGGTGGGGGGG	186131	3' end (non-template strand +29)
Between VOC family protein and MutL gene, *voc* (DR_1695) and *mutL* (DR_1696)	1715886–1716312	GGGGATAGGGGTTTGGGGTTGGGG	1716191	Intergenic
Between the DnaN and DnaA genes, *dnaN* (DR_0001) and *dnaA* (DR_0002)	1178–1902	GGGGGGTTATCCACAGGGCATTTTTAGGG	1638	Intergenic
Between cation: proton antiporter and miaB, *cpa* (DR_1571) and *miaB* (DR_1572)	1160742–1161327	GGGGTAAGGGGCTGGGGGATGGGG	1161171	Intergenic
Between the ABC-F family ATP-binding cassette and DUF2087 genes, *abc-F* (DR_1103) and *duf* (DR_1104)	1113653–1114067	GGGGTAAGGGGCTGGGGGATGGGG	1113954	Intergenic
RecA (DR_2340) (positive control)	2337795–2338886	GGTCGGGGCGGCGGTGG	2335628	Positive
HS (negative control)	–^*a*^	TCCTGCATCTTCAGGC	**–**	Negative

^*a*^ “–” indicates that the HS sequence is not part of the *D. radiodurans* genome. It is used as a negative control, and therefore, gene position and G4 motif location are not applicable.

### Validation of *in vitro* G4 structure formation in the presence of various salts

#### ThT fluorescence enhancement assays

To check the G4 structure formation, the synthesized PQS (5 µM) were heated at 95°C in the presence of KCl (50 or 100 mM) or MnCl_2_ (0.1 mM, 2 mM, or 10 mM), or in combinations along with 10 mM Tris buffer (pH 7.4) and slowly cooled down to room temperature. These annealed sequences were mixed with 0.5 µM ThT in a 96-well microplate. The fluorescence spectra were collected between 440 and 700 nm after excitation at 425 nm in a Synergy H1 Hybrid multi-mode microplate reader. The fluorescence intensity of the ThT alone or in the presence of PQS was compared. To quantify the ThT fluorescence enhancement, the ratio of fluorescence intensity of ThT when an oligonucleotide is present (FI) to the intensity of ThT in the presence of buffer (FI_0_) after removing the background signal from the buffer solution was calculated and plotted. The fluorescence emission spectra of ThT were plotted using GraphPad Prism 8.0. The fold change in fluorescence enhancement was calculated as described earlier (25).

#### Circular dichroism spectroscopy

Circular dichroism (CD) spectroscopy was performed to examine the effect of various salts on G4 DNA topologies *in vitro*, as discussed earlier (26). In brief, PQS (8–10 µM) were annealed in the presence of different monovalent cations such as KCl (100 mM), NaCl (50 mM), LiCl (50 mM), and divalent cations MgCl_2_ (10 mM) and MnCl_2_ (0.1, 2, and 10 mM) along with 10 mM Tris buffer (pH 7.4) by heating at 95°C for 10 min and cooling down to room temperature. CD spectra were recorded on a spectrophotometer (Biologic MOS-500 CD spectropolarimeter), in the wavelength range from 220 to 320 nm with a scanning speed of 100 nm/min and a response time of 2 s, using a quartz cuvette with a path length of 1.0 mm. The experiments were repeated in triplicate, and the spectrometer’s software was used to subtract the buffer contribution to the CD characteristics. Parallel structures display a maximum absorbance at approximately 264 nm and a minimum at 240 nm, while antiparallel structures exhibit a maximum at 290 nm and a minimum at 260 nm. Hybrid structures present a composite profile with maxima at both 290 nm and 264 nm and a minimum at 240 nm (27).

### RNA isolation and real-time qPCR analysis

The total RNA was isolated according to the previously described protocol (28). In brief, the cells were cultured in TGY broth with or without G4 ligand (100 nM NMM) and subjected to unirradiated or irradiated (1 h PIR) conditions. The cell pellet containing ∼3  ×  10^8^ cells, preserved in TRIzol reagent, was thawed, and the cells were lysed by vigorous vortexing. The suspension was treated with chloroform and incubated at room temperature for 15 minutes, followed by centrifuging at 12,000  ×  *g* at 4°C. The upper aqueous layer containing RNA was separated and precipitated by adding isopropanol. The pellet was dissolved in diethyl pyrocarbonate-treated RNase-free water and further treated with DNase. The purity of the RNA (OD_260_/OD_280_ ~ 2) was assessed using the Synergy H1 hybrid multimode microplate reader. Until further use, the RNA was snap-frozen in liquid nitrogen. To validate the expression levels of putative genes, real-time qPCR (qPCR) analysis was performed as described earlier (29). In short, total RNA (~20 nanograms) taken from these cells, along with gene-specific primers, was examined using a LUNA one-step RT-qPCR kit or Takara one-step PrimeScript RT-PCR Kit II following the manufacturer’s instructions. Reverse transcription was performed at 55°C for 10 minutes, followed by an initial denaturation at 95°C for 1 minute. Subsequently, 40 PCR cycles were performed, each cycle including denaturation at 95°C for 10 seconds and extension at 60°C for 30 seconds. A melting curve was generated from 60 to 95°C, starting with a 90 second hold at 60°C and increasing by 1°C/second using a BioRad CFX Duet Real-Time PCR System. The data obtained were analyzed using accompanying software with 16S rRNA as a control. All the gene-specific primers used are listed in Table S2 at https://barc.gov.in/publications/mBio01639-25R1/index.html. The data represent the fold change (mean ± SEM) in gene expression from three independent experiments, and statistical significance was analyzed using Student’s *t*-test.

### Cell survival studies

*D. radiodurans* R1 cells in exponential phase grown at 32°C in TGY medium in the presence or absence of ligands (100 nM of NMM or 1.5 µM TMPyP4) were maintained throughout the experiments as per the requirement. These cells were exposed to 6 kGy gamma radiation, and the dose was delivered at a dose rate of 3.75 kGy/h over 1.6 hours (Gamma Cell 5000, 60Co., Board of Radiation and Isotopes Technology, DAE, Mumbai, India). Cells were exposed to other DNA-damaging agents like H_2_O_2_ (70 mM and 100 mM), UV rays (1,000 J/m^2^), and mitomycin C (MMC; 5 µM) in the presence and absence of NMM or TMPyP4. After treatment, the cells were pelleted and diluted to 20-fold in fresh TGY medium, with or without NMM or TMPyP4, and the growth was monitored at 600 nm in a Synergy H1 multimode plate reader for 18 h. The growth curve data were then analyzed using GraphPad Prism 8.0 software and plotted. The linear growth rate of cells treated with different DNA-damaging agents was calculated using the bacterial growth rate formula μ = (ln *N*_*t*_ − ln *N*_0_)/(*t* − *t*_0_), where μ = bacterial growth rate, *N*_t_ = total bacteria number at the time (hour), *N*_0_ = total bacteria number at the time *t*_0_, and *t* − *t*_0_ = time period of calculating the growth rate. For the spot plate assay, the deinococcal cells grown with or without NMM and TMPyP4 were subjected to various DNA-damaging agents as described above. The cultures were diluted to appropriate serial dilutions, and 2 µL was spotted onto TGY agar plates, with or without NMM or TMPyP4 supplementation. Growth was monitored after 30–36 h of incubation at 32°C.

### Microscopic studies

#### ThT staining

*In vivo* formation of G4 structures was monitored by staining with ThT. *D. radiodurans* R1 cells were grown overnight at 32°C in TGY medium and exposed to various DNA-damaging agents as discussed earlier. After treatment, the cells were allowed to recover with constant shaking and collected at 1 h and 4 h post-treatment. The *recQ* mutant (Δ*recQ*) (30) of *D. radiodurans* cells was also grown overnight at 32°C in kanamycin (8 µg/mL) containing TGY medium. All the cells were treated with various DNA-damaging agents, and ThT staining was performed to visualize the *in vivo* formation of G4 structures. The cells were washed in phosphate-buffered saline (PBS) and stained with 2 µg/mL 4′,6-diamidino-2-phenylindole, dihydrochloride (DAPI) for nucleoid staining and 2 µM ThT for G-quadruplex visualization. These cells were washed with PBS to remove excess dye and resuspended in a small volume of PBS. 2–3 µL of cell suspension was dropped on a glass slide coated with 0.8% agarose and covered with glass cover slips. The confocal microscopy was performed using an Olympus IX83 inverted microscope as described earlier (31). The DAPI fluorescence was visualized using a filter having 460 nm emission when excited at 402 nm. The ThT fluorescence was monitored at an excitation wavelength of 438 nm, and emission was recorded at 560 nm using the fluorescein isothiocyanate (FITC) channel. Image analysis was carried out using the built-in CellSens software. Approximately 200–250 cells from three independent experiments were examined for quantitative analysis, and data were plotted using GraphPad Prism 8.0.

#### Immunofluorescence microscopy

The formation of G4 structure *in vivo*, detection of ssDNA, and levels of 8-oxoG during PIR were checked using an anti-G4 antibody (clone 1H6), anti-BrdU antibody, and anti-8-Oxoguanine antibody, respectively, as described earlier (32–34). Briefly, *D. radiodurans* wild type (R1) treated and untreated with NMM were subjected to 6 kGy radiation and were recovered with fresh medium. PIR samples were collected at regular time intervals for *in vivo* G4 structure formation and detection of ssDNA. After collection, the cells were fixed with 4% paraformaldehyde solution and then washed in PBS. Furthermore, the cells were permeabilized by treatment with lysozyme (2 mg/mL) for 1 h at 37°C, then with 0.1% Triton X-100 in PBS at 37°C for 5 min. The cells were washed with PBS and treated with 40 µg/mL RNase A for 30 min at 37°C. The cells were again washed with PBS and resuspended. Around 5 µL was spread onto a poly-l-lysine pretreated slide and allowed to air dry. Fixation was done by treating the slides with 4% PFA at 37°C for 30 min. Using a blocking solution of 2% BSA in PBS-T (0.05% Tween 20 in PBS), samples were blocked for 2 hours at 37°C and subsequently incubated with the G4 antibody overnight in the same solution. Cells were washed with PBS-T twice for 20 min and then incubated with a secondary antibody, anti-mouse IgG conjugated with Alexa Fluor 594 (Sigma) for 2 h in a blocking solution. Cells were again washed with PBS-T for 20 min twice, and slides were mounted using a mounting medium containing antifade DAPI. Cells were visualized under a microscope, and the fluorescent intensity profile of ~200 cells was analyzed using automated Olympus CellSens software and plotted using GraphPad Prism 8 software. Similarly, to detect ssDNA, an immunofluorescence assay using an anti-BrdU antibody was performed. For this, *D. radiodurans* wild type (R1) treated with or without NMM was subjected to 6 kGy radiation and recovered in fresh medium for 1 h. Then, the cells were pulse-labeled with BrdU for 15 min and transferred to a fresh medium containing NMM-TGY and TGY alone with the addition of thymidine. The same was done with 4 h samples. After 30 minutes of thymidine addition, the cells were collected. The cells were then fixed and permeabilized as described earlier. To denature the DNA, the cells were treated with 1 M HCl for 30 min at room temperature, and later, the cells were washed twice with 1× PBS. The microscopic studies were done as described above. For the detection of 8-oxoguanine after PIR, the cells treated with or without NMM were exposed to 6 kGy radiation, and samples were collected after 15 min and 1 h. The cells were fixed and permeabilized. Furthermore, they were incubated with anti-8-Oxoguanine antibody overnight in a blocking solution and processed further as described above.

To visualize the *in vivo* formation of G4 structures in Δ*recQ*, an anti-DNA G4 antibody (clone 1H6) was used as described for wild-type cells. For morphological studies, both the wild-type and Δ*recQ* cells were grown, washed with PBS, stained with DAPI, and visualized. Approximately 200 cells were analyzed using Olympus CellSens software to quantify various cell morphologies. The resulting data were plotted with GraphPad Prism 8.0 software, and differences were considered significant if the *P*-value was below 0.05 (95% confidence interval).

### Pulsed-field gel electrophoresis

The repair of damaged DNA is monitored by pulsed-field gel electrophoresis (PFGE), as described earlier (35). Briefly, cells were grown with and without NMM, resuspended in a fresh medium, and were irradiated with a dose of 6 kGy. The cells were diluted in TGY and allowed for PIR, and samples were collected at different time intervals. Each collected aliquot was pelleted by centrifugation and resuspended in butanol-saturated PBS followed by another centrifugation step. The obtained pellets were further resuspended in 0.5 M ethylenediaminetetraacetic acid (EDTA) and kept at 60°C for 30 min, then pelleted and resuspended in Tris-EDTA buffer. A cell suspension was mixed in a 1:1 volume/volume ratio with 1.5% low-melting-point agarose and solidified into plugs using a mold. These plugs were incubated overnight with lysis solution containing 1 mg/mL of lysozyme at 37°C and later incubated at 50°C in 1% sarcosyl and 1 mg/mL proteinase K solution for overnight. The plugs were subjected to PFGE in 0.5× TBE using a CHEF-DR III electrophoresis system (Bio-Rad) at 6 V/cm^2^ for 16 h at 14°C, with a linear pulse ramp of 10–60 s and a switching angle of 120°. The gel was stained with ethidium bromide for visualization and documentation.

### Statistical analysis

For all the statistical analyses, the Student’s *t*-test was used. The significance *P* values obtained at 95% confidence intervals are depicted as **** for *P* value < 0.0001, *** for *P* value < 0.001, ** for *P* value of 0.05–0.001, and * for *P* value < 0.05.

## RESULTS

### PQS fold into different topologies

**Fig 1:**
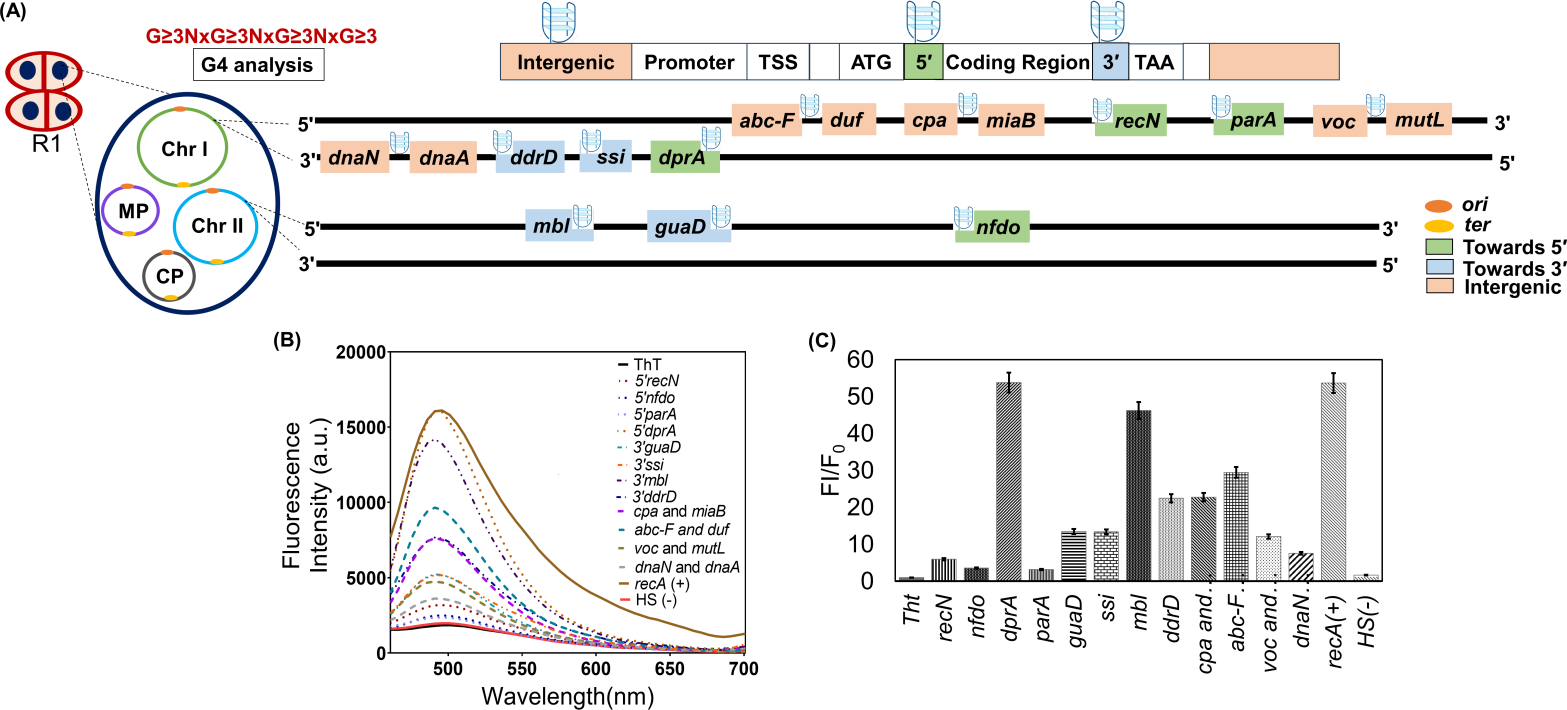
*In silico* analysis and *in vitro* validation of PQS using ThT fluorescence assay. (**A**) Schematic representation of PQS present at various positions (5′, 3′, and intergenic region) of the *D. radiodurans* genome investigated in this study. (**B**) Fluorescence emission spectra of ThT in the presence of monovalent cation KCl (100 mM) alone and in the presence of synthesized PQS. The fluorescence spectra were collected between 440 and 700 nm after excitation at 425 nm. The experiment was performed in triplicate. (**C**) The bar graph represents the fold change in fluorescence enhancement of ThT, and error bars correspond to 95% confidence intervals. A non-G4-forming sequence (HS) served as a negative control, while *recA* was used as a positive control.

Previously, the presence of PQS at the upstream regions of most DNA repair genes in *D. radiodurans* was reported (20). However, not much has been explored about the PQS in regions other than those near the promoter. Here, the existence of PQS at different positions within genes (toward the 5′ or 3′ ends) or in intergenic regions was investigated as described in methodology (Fig. 1A). The analysis revealed the occurrence of 3G run sequences at different genomic locations on chromosome I, chromosome II, megaplasmid, and small plasmid in this bacterium (see Tables S3 to S6 at https://barc.gov.in/publications/mBio01639-25R1/index.html). For further study, we have taken the genes that contain the PQS within ~200 bp of the 5′ or 3′ end of the genes. Genes such as *recN*, *dprA*, *nfdo*, and *parA* contain 3G run sequences toward their 5′ end. In contrast, genes such as *ddrD*, *ssi*, *guaD,* and *mbl* contain PQS towards their 3′ end. Additionally, some PQS were identified in intergenic regions between *voc* and *mutL*, *dnaN* and *dnaA*, *cpa* and *miaB*, and *abc-F* and *duf* (Fig. 1A; Table 1). The above-identified genes encode proteins that function in various cellular pathways, as mentioned in Table S7 at https://barc.gov.in/publications/mBio01639-25R1/index.html (17, 36–50). In addition to these deinococcal PQS, a non-G4-forming DNA sequence (HS) and a known deinococcal G4-forming sequence (*recA*) were taken as the negative control and positive control, respectively (21). Further studies were carried out to check the G4 structure formation by the PQS in the presence of different salts.

The formation of G4s was checked by the ThT fluorescence assay as described in the methodology. ThT is a benzothiazole that becomes fluorescent in the presence of the G4 structures (25). Previous research demonstrated that ThT can be utilized to specifically detect human telomeric G4 structures, effectively distinguishing them from duplex or ssDNA (51). For G4 detection, the fluorescence intensity of the ThT in the presence of buffer and with the PQS was compared. ThT exhibited an emission maximum at approximately 490 nm. Across all tested sequences, as mentioned in Table 1, fluorescence enhancement was noticed in the presence of PQS (Fig. 1B). Additionally, ThT fluorescence enhancement was observed in the positive control *recA,* while no significant increase in ThT fluorescence was observed with HS, the negative control. The results also indicated that all the PQS led to an approximately or >5-fold increase in ThT fluorescence compared to both the non-G4 sequence and ThT alone. Few PQS, such as *dprA*, *mbl*, and *recA* sequences, exhibited over a 40-fold fluorescence enhancement (Fig. 1C). Thus, the ThT fluorescence assay results suggest that PQS may fold into stable quadruplex structures *in vitro*.

Previously, the studies on the folding kinetics of telomere G4s in the presence of monovalent cations noticed that with K^+^ (50 mM), complete G4 folding was achieved without any ssDNA, but with Na^+^ (150 mM), some extent of ssDNA remained (52). K^+^ was also shown to replace Na^+^ from a stable G4 fold (53). In general, Li^+^ cannot support G4 folding because of its smaller size but can influence or promote the folding fraction differentially with K^+^ or Na^+^. Building upon these established findings regarding the influence of different metal ions on G4 structure, the CD was performed to check the distinct topologies obtained by deinococcal G4s with various metal ions. The CD spectra of G4 DNA are indicative of their topology, as mentioned in the materials and methods section. The CD results revealed that in the presence of KCl, all the examined PQS were stabilized and formed a parallel topology, regardless of their positions, at least *in vitro*. The presence of NaCl resulted in distinct topologies: parallel in the case of 3′-*guaD*, 3′-*mbl,* and 5′-*dprA;* hybrid in the case of 3′-*ssi*, 5′-*recN,* and *nfdo*; and antiparallel in the case of 3′-*ddrD* and intergenic between *voc* and *mutL, cpa* and *miaB*. All PQS were destabilized in the presence of LiCl, except for the one located in the intergenic region between *cpa* and *miaB*, which showed antiparallel topology (see Fig. S1 at https://barc.gov.in/publications/mBio01639-25R1/index.html).

**Fig 2:**
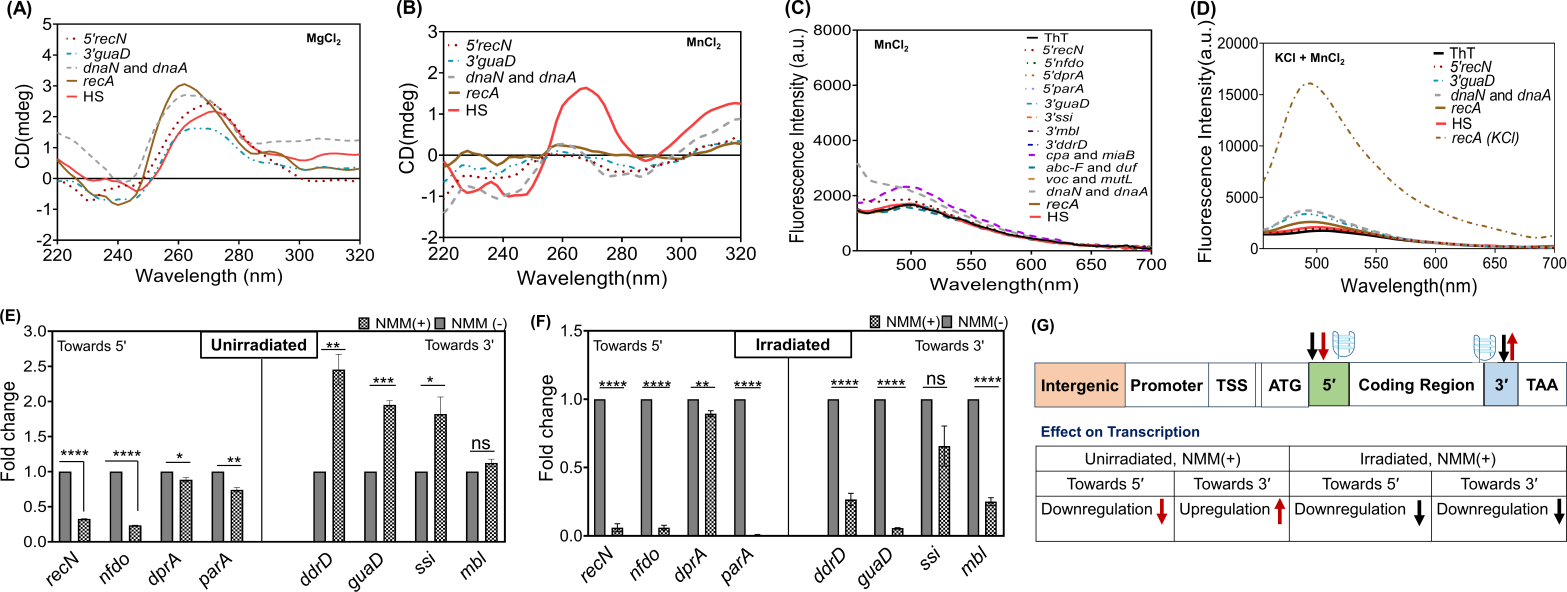
Effect of divalent cation on the topology of PQS and their positional effect on gene expression. (**A**) CD spectra of synthesized PQS in the presence of 10 mM MgCl₂. The solid line indicates the controls. (**B**) CD spectra of synthesized PQS in the presence of 10 mM MnCl₂. (**C**) The fluorescence emission spectra of ThT in the presence of 10 mM MnCl₂. (**D**) The fluorescence assay was performed in the presence of both KCl (100 mM) and MnCl₂ (0.1 mM) simultaneously. The *recA* (KCl) represents the fluorescence emission spectra only in the presence of KCl (100 mM). (**E**) Real-time PCR analysis to check the positional effects of native G4s on endogenous gene expression. *D. radiodurans* cells were grown in TGY medium with or without NMM in an unirradiated condition and (**F**) irradiated condition. The acquired data were processed using GraphPad Prism 8, and gene expression levels were presented as fold changes relative to the 16S rRNA as a control. Data represented in the graph are the mean ± SE (*n* = 3), and the statistical significance was assessed with Student’s *t*-test. The *P*-values attained at 95% confidence intervals are depicted as * for <0.05, ** for <0.01, *** for <0.001, **** for <0.0001, and ns for non-significant (>0.05). (**G**) Schematic representation of the positional influence of G4 motifs on gene expression. The red arrow indicates the effect on gene expression under unirradiated conditions when stabilized with NMM, while the black arrow represents the effect under irradiated conditions.

Earlier studies showed that certain divalent cations like Mn^2+^, Co^2+^, or Ni^2+^ can disrupt the G4s folding even in the presence of K^+^ (54). *D. radiodurans* cells possess high levels of divalent cation Mn^2+^ (55; 56). Thus, considering the physiological relevance of Mn^2+^ in this organism, the *in vitro* formation of some G4 structures was also assessed in the presence of divalent cations, such as Mg^2+^ and Mn^2+^. All of the PQS checked (3′-*guaD*; 5′-*recN*; intergenic between *dnaN* and *dnaA; recA*) formed stable parallel structures in the presence of Mg^2+^ (Fig. 2A). However, surprisingly, all of them were destabilized when annealed in the presence of Mn^2+^ (10 mM; Fig. 2B and C). Even at lower concentrations of Mn^2+^ (0.1 mM and 2 mM), stable G4s were not formed (see Fig. S2B and D at https://barc.gov.in/publications/mBio01639-25R1/index.html). The ThT fluorescence enhancement assay also revealed that in the presence of Mn^2+^, all the G4s were destabilized, as no increase in ThT fluorescence was observed. In contrast, the same control sequence, *recA*, in the presence of KCl showed fluorescence enhancement (Fig. 2D; see S2A and C at https://barc.gov.in/publications/mBio01639-25R1/index.html). This indicates that different metal cations exhibit differential effects on the PQS of *D. radiodurans*.

Next, the effect of the simultaneous presence of KCl (100 mM) and MnCl_₂_ (0.1 mM) on G4 folding was checked by ThT fluorescence assay. As shown in Fig. 2D, fluorescence enhancement was observed with the KCl control; however, when both metal ions were present, fluorescence enhancement was absent for all the PQS, indicating that MnCl_₂_ has an overall destabilizing effect on G4 folding. All these results suggest that deinococcal G4s adopt different topologies in the presence of monovalent and divalent cations, and despite having the same sequence, they can adopt different topologies depending on the metal cations in the surroundings.

### G4s regulate gene expression differently depending on their location

A systematic study in *E. coli* demonstrated that G4s in the promoter region or at the 5′-UTR on the antisense strand increase transcription while those at the 5′-UTR on the sense strand decrease transcription (57). However, G4 presence in the 3′ UTR region has no significant effect. In *D. radiodurans,* to investigate the positional influence of native G4s present at 5′ or 3′ ends, the transcript levels were checked under both G4-stabilizing and non-stabilizing conditions, with and without gamma radiation treatment. RT-qPCR results indicated that these G4 motifs differentially regulate transcription. Under normal growth conditions, in the presence of NMM, the expression of transcripts containing G4 motifs at the 5′ end, such as *recN*, *nfdo*, *dprA,* and *parA*, was downregulated (Fig. 2E). The G4 sequences present in the *recN, nfdo,* and *parA* genes are located on the non-transcribed strand, while in the *dprA gene,* they are located on the transcribed strand. The observed effect may be due to the interference of stable G4 structures in transcription re-initiation or elongation. In contrast, transcripts having G4 motifs at the 3′ end of genes, including *ddrD*, *guaD,* and *ssi,* were upregulated when compared to control samples. This increase could be due to the stable G4s at the 3′ end in facilitating the recruitment of RNA polymerase to ssDNA. However, for *mbl,* which has a 3′ G4 motif, no significant change was observed (Fig. 2E). Interestingly, after gamma radiation treatment, the presence of NMM resulted in downregulation of genes that contain native G4s either at 5′ or 3′ ends of the genes (Fig. 2F). An exception was the *ssi* gene, which showed no significant change. This reduction in gene expression after irradiation in the presence of NMM could result from the increased G4 structure formation in the ssDNA generated during the repair process. Another possibility might be NMM, a G4-specific ligand that may also compete with other cellular factors for the same binding site, resulting in altered G4 dynamics and gene expression (58). Moreover, an analysis of previously reported transcriptome sequencing (RNA-Seq) data corroborated these results. After irradiation, normal growing cells showed increased fragments per kilobase of transcript per million mapped reads (FPKM) values for the DNA repair genes that contain PQS in their promoter, whereas the cells treated with NMM exhibited lower FPKM for the same genes, indicating a decrease in the transcript levels (21) (see Fig. S3A through H at https://barc.gov.in/publications/mBio01639-25R1/index.html). This suggests that the arrest of G4 dynamics affects the gamma radiation-responsive gene expression. Furthermore, RT-qPCR was done to validate, and the result suggests that in the absence of NMM post-irradiation, there was an increase in the transcript levels of DNA repair genes examined in this study, such as *ddrD*, *recN*, and *dprA,* as reported in earlier findings (see Fig. S3I at https://barc.gov.in/publications/mBio01639-25R1/index.html) (59). Thus, both RNA-Seq and RT-qPCR results suggest that the native G4 motifs located at the 5′ or 3′ ends within the genes have differential effects on endogenous gene expression levels when assessed at the genomic level in this bacterium (Fig. 2G). To understand the molecular effect of gamma radiation on these secondary structures, the *in vivo* G4 structure formation was investigated.

### *In vivo* G4 structure formation and dynamics in response to cellular stresses

**Fig 3:**
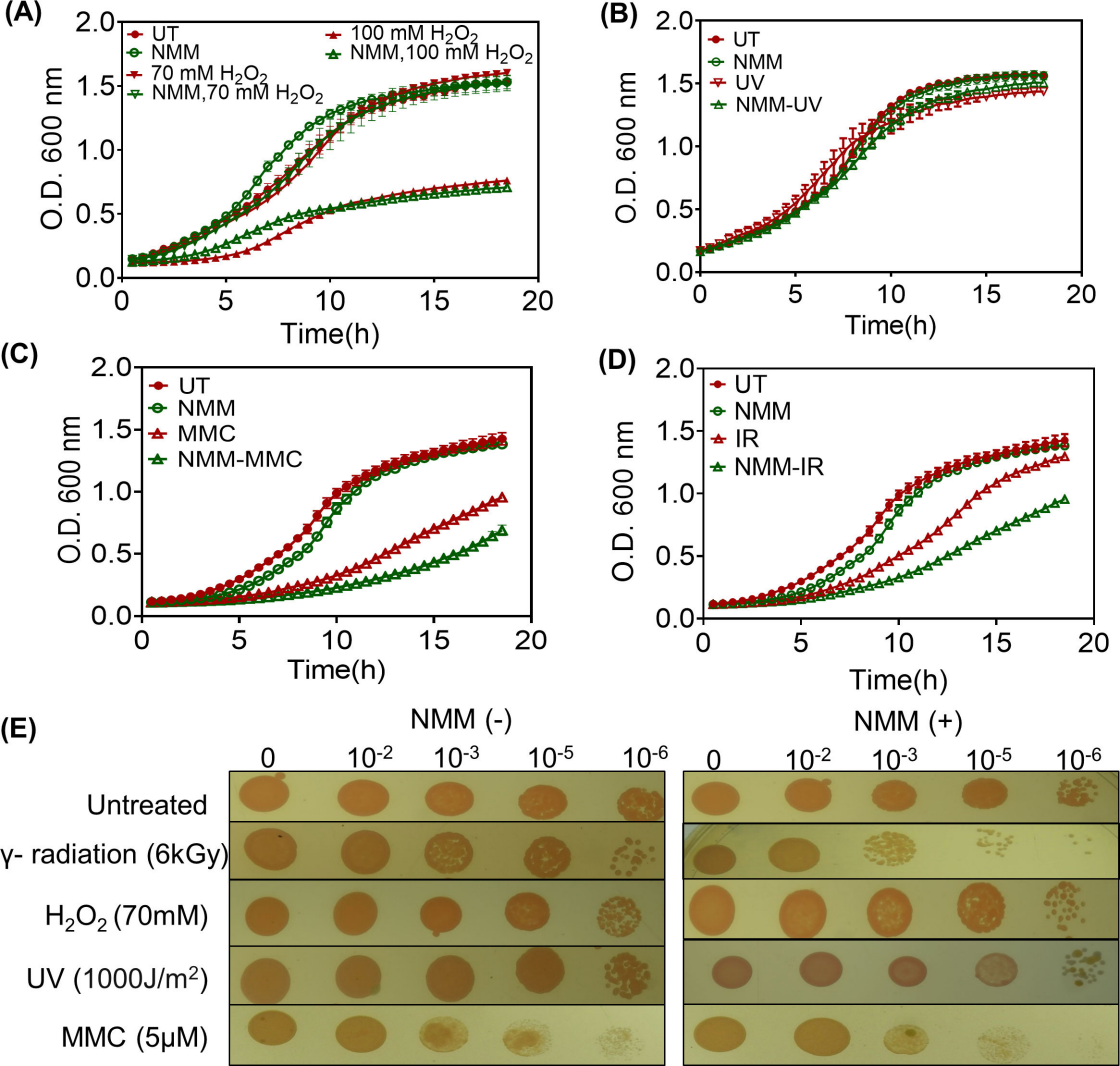
Effect of various DNA-damaging agents on growth kinetics in the presence of G4-binding ligand, NMM. (A to D) Effect of (**A**) hydrogen peroxide (H_2_O_2_) (70 mM and 100 mM), (**B**) UV rays (1,000 J/m^2^), (**C**) mitomycin C-MMC (5 µM), and (**D**) γ-radiation (6 kGy) on growth kinetics in the presence and absence of NMM. Data are represented here as the mean ± SEM of three independent experiments. (**E**) Spot plate assay of wild-type cells grown in the presence of ligand (NMM+) and absence of ligand (NMM−) was exposed to different stresses. Different dilutions were spotted on TGY and TGY containing NMM agar plates. A delayed growth was observed in response to MMC and γ-radiation-treated cells in the presence of NMM.

*D. radiodurans* cells subjected to 6 kGy radiation exhibit sensitivity to gamma radiation in the presence of G4 DNA-stabilizing ligands (19, 21). In addition to gamma radiation, *D. radiodurans* is known for its resistance to other DNA-damaging agents such as UV radiation, H_2_O_2_, and mitomycin C. To investigate whether G4s play any role in cell survival against these DNA-damaging agents or not, growth was analyzed. Growth studies indicated that in the presence of G4 ligands, no changes were observed when the cells were subjected to H_2_O_2_ (Fig. 3A; see Fig. S4A at https://barc.gov.in/publications/mBio01639-25R1/index.html) and UV stresses (Fig. 3B; see Fig. S4C), while the cells showed sensitivity to MMC treatment (Fig. 3C; see Fig. S4E) and gamma radiation (Fig. 3D; see Fig. S4G), as reported earlier (21). The calculated growth rate showed no significant changes in growth rate with H_2_O_2_ and UV treatments in the presence of NMM or TMPyP4 (see Fig. S4B and D at https://barc.gov.in/publications/mBio01639-25R1/index.html). But a significant decrease in the growth rate was observed in response to MMC treatment (see Fig. S4F at https://barc.gov.in/publications/mBio01639-25R1/index.html) and ionizing radiation (see Fig. S4H) in the presence of NMM and TMPyP4. The cell survival analysis using a spot plate assay was also conducted, and the results revealed that G4 ligands, NMM and TMPyP4, sensitize the *D. radiodurans* cells to ionizing radiation and mitomycin C-mediated DNA damage (Fig. 3E; see Fig. S4I at https://barc.gov.in/publications/mBio01639-25R1/index.html). Mitomycin C induces interstrand DNA crosslinks, which lead to double-strand breaks (DSBs). The potential for DSBs to initiate G4 formation as a signal or to assist in the DNA damage repair process cannot be dismissed.

**Fig 4:**
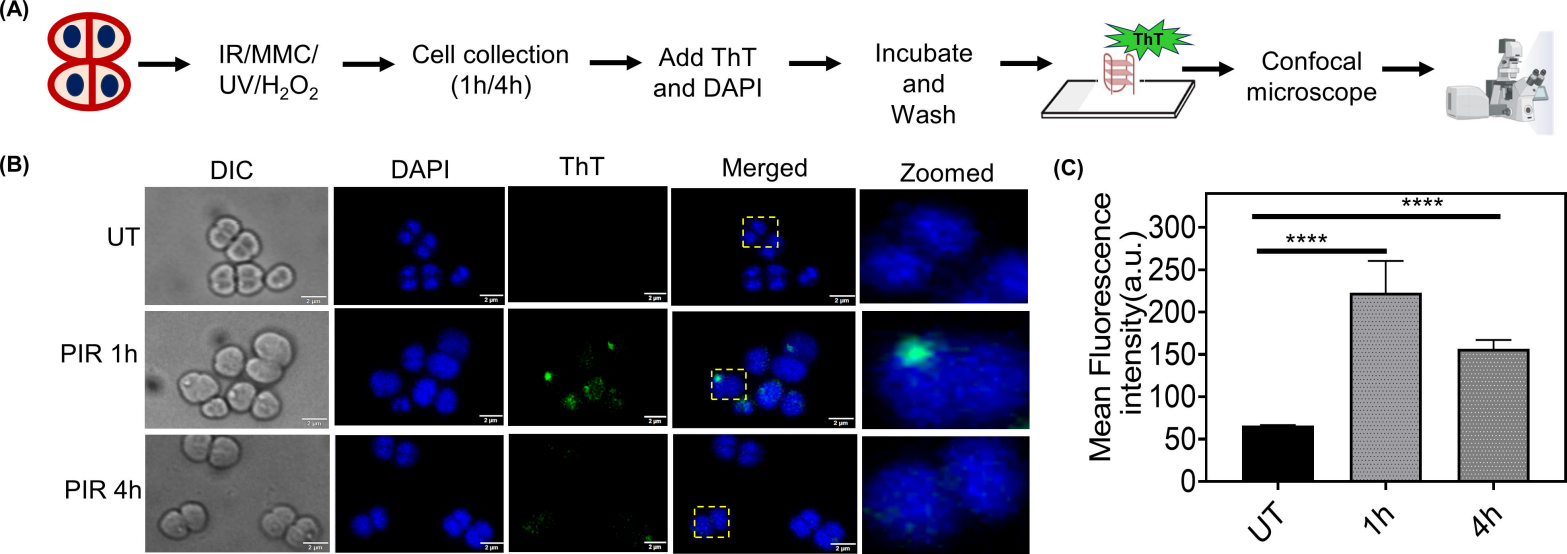
*In vivo* G4 structure dynamics during PIR using ThT. (**A**) Schematic representation of the experimental setup employed for fluorescence microscopy using ThT dye. (**B**) Fluorescence microscopic examination of *D. radiodurans* cells stained with ThT after gamma radiation. At 1 h and 4 h PIR, samples were collected and stained with ThT to visualize the G4 structure, and UT represents the unirradiated sample. Images were taken in differential interference contrast (DIC), DAPI (for nucleoid), FITC (for ThT), and merged channels for DAPI and FITC. The scale bar is 2 µm. The images shown here are representative pictures of the experiments conducted at least three times. (**C**) The mean fluorescence intensity was calculated using the built-in Cell Sense software, and statistical analysis of post-irradiated samples, 1 h and 4 h, was compared to UT cells, done in 200–250 cells, and plotted. The *P*-values attained at 95% confidence intervals are depicted as **** for <0.0001.

Next, the *in vivo* formation of G4s in *D. radiodurans* was checked using ThT staining and immunofluorescence experiments, as described in the materials and methods section. ThT binds to stable G4 DNA structures, resulting in a significant increase in fluorescence that can be detected using the FITC channel under a microscope (Fig. 4A). As shown in Fig. 4B, cells grown under normal conditions (UT) exhibited minimal ThT intensity, suggesting rapid dynamics or rare formation of these secondary structures. However, after gamma radiation treatment at 1 h PIR, the intensity of ThT increased, and a few prominent high-intense foci or accumulations of ThT were observed. At 4 h PIR, the mean fluorescent intensity of ThT decreased compared to 1 h PIR (Fig. 4C). This suggests that during the early period of PIR, a higher number of G4 structures form or cluster in the cells, which may subsequently resolve at later stages of PIR due to their dynamic nature. G4 formation in cells treated with other stresses was also observed. Small dispersed foci of ThT at 1 h were observed in cells treated with UV and H_2_O_2_, with a reduction in the number of such foci at 4 h. In contrast, following MMC treatment, a few dispersed foci were still observed even at 4 h (see Fig. S5A and B at https://barc.gov.in/publications/mBio01639-25R1/index.html). Nevertheless, under all observed stress conditions, cells at 1 h PIR showed the highest intensity or accumulation of ThT foci.

**Fig 5:**
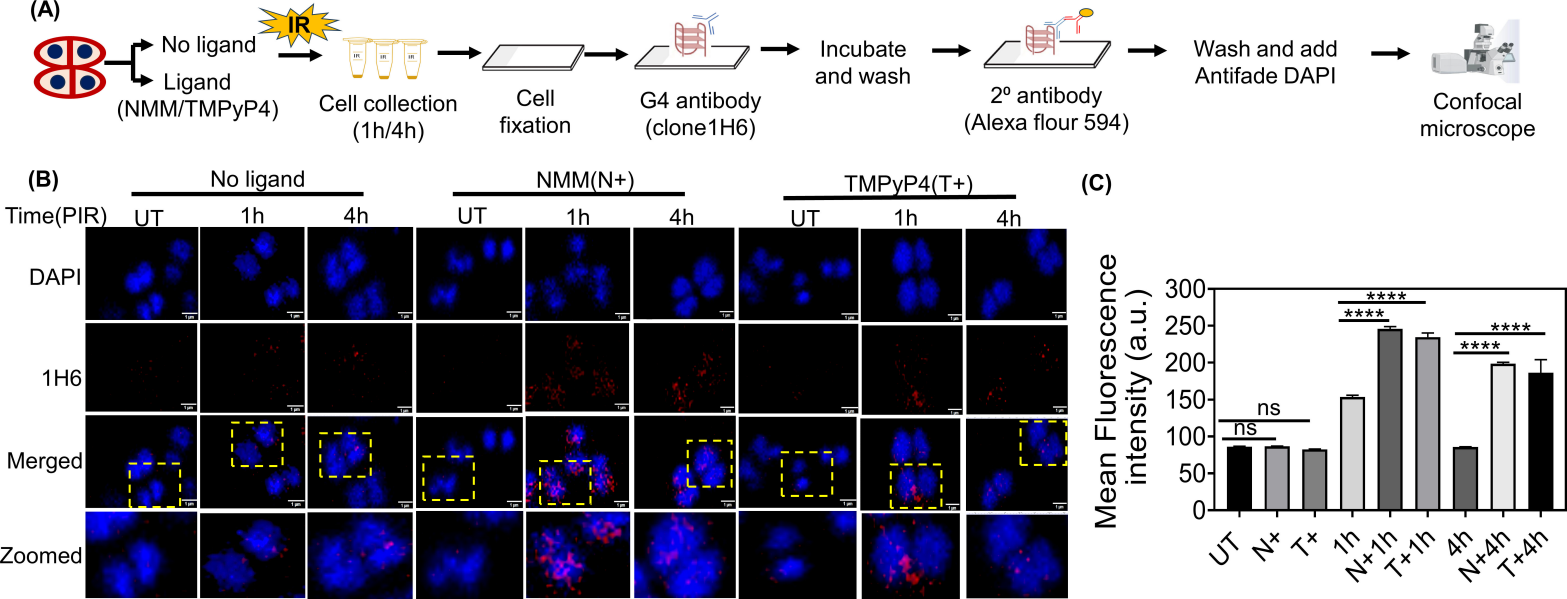
*In vivo* formation of G4 structures and their dynamics during PIR. (**A**) Schematic representation of the experimental set-up employed for immunofluorescence microscopy analysis using an anti-G-quadruplex antibody. (**B**) Microscopic images of *D. radiodurans* cells subjected to gamma radiation (6 kGy) in the absence of G4 ligand (No ligand) or the presence of G4 ligands [NMM*(*N*+*) and TMPyP4(T+)] and PIR cells at 1 h and 4 h (PIR 1 h and 4 h) for visualization of *in vivo* G4 structure formation using G4 structure-specific antibody (clone 1H6). UT represents the unirradiated cells grown in the absence or presence of respective ligands. The microscopic images were taken in different channels, DAPI (for nucleoid), tetramethylrhodamine isothiocyanate (TRITC; for G4 formation), and merged channels for DAPI and TRITC. The zoomed panel represents the image of individual cells for better visualization (scale bar is 1 µm). The representative images are of biological triplicates. (**C**) The mean fluorescence intensity was calculated using automated CellSens software, and graphs were plotted using GraphPad Prism, and statistical analysis was done in 100–150 cells using Student’s *t*-test. The *P*-values obtained at 95% confidence intervals are indicated as **** for <0.0001 and ns (non-significant) for >0.05.

Furthermore, to monitor the formation of G4 structures *in vivo* and assess the effect of G4-binding ligands, NMM and TMPyP4, on their dynamics during PIR, immunofluorescence was performed using an anti-G-quadruplex (1H6) antibody as described earlier (Fig. 5A). All the samples (NMM or TMPyP4 treated as well as untreated) were subjected to gamma radiation and examined (Fig. 5B). The microscopic results indicated that 1 h PIR samples exhibited a stronger fluorescence signal compared to untreated samples (UT), suggesting the *in vivo* formation of G4 structure following gamma radiation treatment. The presence of NMM (N*+*) and TMPyp4 (T+) at 1 h post-irradiation further increased the fluorescence signal (1 h N+ and 1 h T+), suggesting that these ligands stabilize G4s in irradiated cells (Fig. 5B and C). At 4 h PIR, NMM- or TMPyP4-treated samples showed a higher signal than untreated samples, indicating sustained G4 stabilization (Fig. 5B and C). This increased mean fluorescence intensity indirectly indicates an arrest of G4 dynamics due to G4 ligand binding. The results thus far suggest that gamma radiation induces more G4 structure formation in the cells and that NMM or TMPyP4 likely interacts with and stabilizes these G4s. This stabilization arrests their dynamics, potentially hindering cell growth and the DNA repair process during post-irradiation, which warrants further investigations.

### Arrest of G4 structural dynamics slows DSB repair kinetics

**Fig 6:**
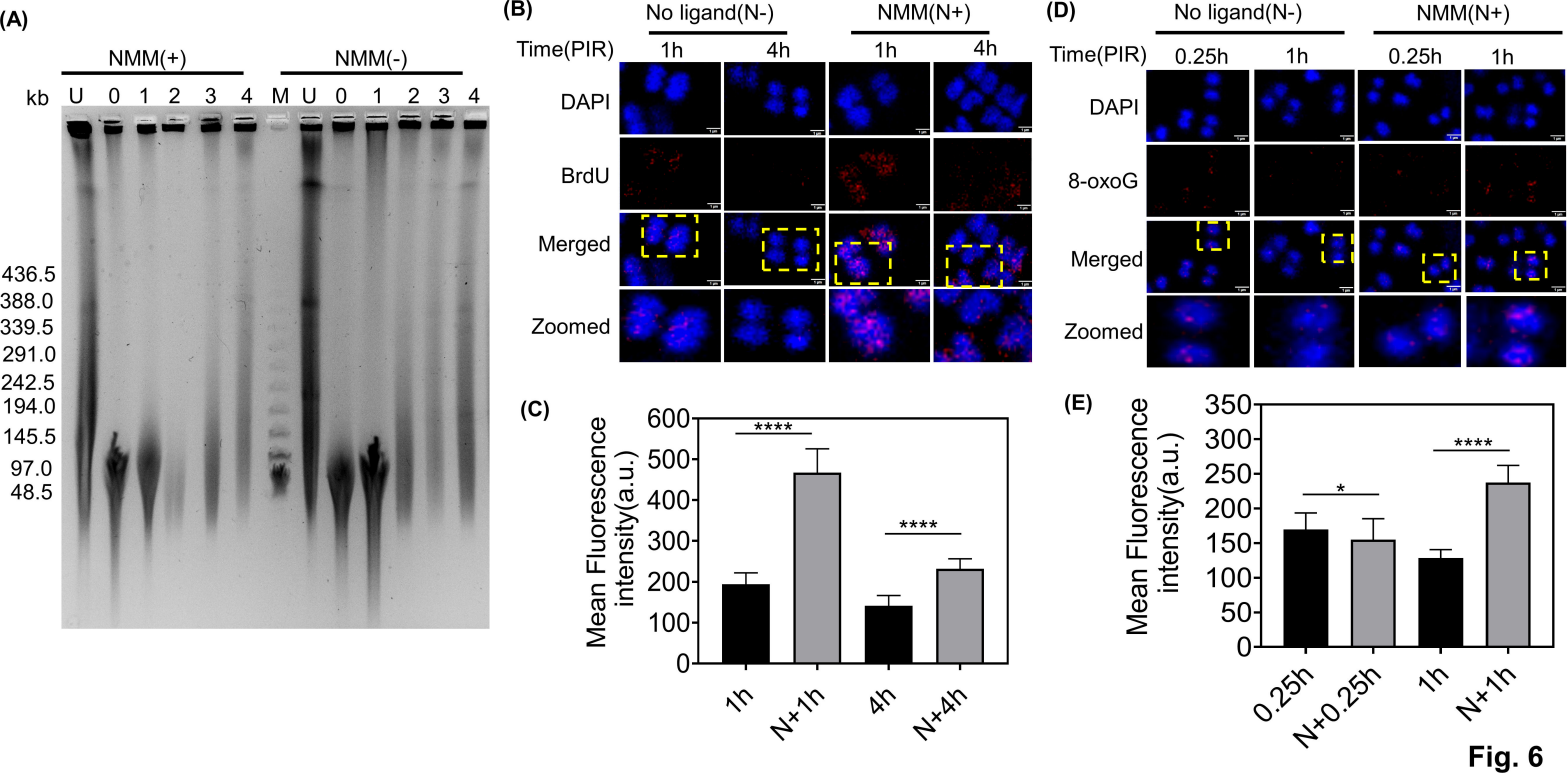
Effect of stabilization of G4 structures on DSB repair in *D. radiodurans*. (**A**) *Deinococcus* cells were grown in the presence (NMM+) and absence (NMM−) of the G4-binding ligand. Gamma radiation untreated (UT) and PIR cells at different time intervals, 0 h, 1 h, 2 h, 3 h and 4 h, were subjected to PFGE along with Lambda PFG marker (M) to check the DSB repair kinetics. (**B**) The immunofluorescence assay of the post-irradiated (1 h and 4 h) NMM untreated (No ligand, N−) and treated (NMM, N+) cells was checked for DNA synthesis and conversion of newly synthesized ssDNA to dsDNA using anti-BrdU antibodies. DAPI (for nucleoid), tetramethylrhodamine isothiocyanate (TRITC; for BrdU), and the merged channels are shown (scale bar: 1 µm). The cells were zoomed for better visualization. The images presented here are representative images from experiments conducted at least three times. (**C**) The mean fluorescence intensity was calculated using automated CellS software, and graphs were plotted using GraphPad Prism. The error bar indicates the mean ± SD. (**D**) The levels of 8-oxoG after γ-radiation were checked by immunofluorescence assay using anti-8-oxoG antibody. The level of 8-oxoG was tested after 0.25 h and 1 h in NMM-treated (N+) cells and NMM-untreated (No ligand, N−) cells. NMM-treated (N+) cells had higher levels of 8-oxoG even after 1 h (N+, 1 h) compared to control cells. The microscopic images show the DAPI (for nucleoid), TRITC (for 8-oxoG), and the merged channels. The cells were zoomed in for better visualization. The scale bar is 1 µm. (**E**) The mean fluorescence intensity was depicted using automated CellSens software. The data presented here represent the mean ± standard deviation (SD), and the experiments were conducted thrice. Statistical analysis was performed using Student’s *t*-test, revealing a significant difference (*P < 0.05, ****P < 0.0001).

*D. radiodurans* cells exhibited a higher number of G4s at 1 h PIR. In the presence of NMM or TMPyP4, a further increment was observed. As ESDSA is the primary pathway for DSB repair in this bacterium, the mechanistic relationship between G4 structural changes and the DSB repair kinetics was studied. PFGE is commonly used to monitor DNA damage repair kinetics. Therefore, PFGE was carried out on *D. radiodurans* cells collected at different time intervals following gamma radiation treatment. PFGE analysis revealed that NMM-treated cells exhibited slower DSB repair kinetics compared to the wild type. In untreated cells (NMM −), the recovery of DNA bands began after 2 h post-irradiation, whereas in NMM-treated cells (NMM +), band appearance started after 3 h of PIR (Fig. 6A), suggesting delayed repair in cells that were treated with G4 ligands. Furthermore, it is known that the early PIR cells possess a high density of ssDNA, which eventually converts into double-stranded DNA (dsDNA) as the repair progresses. During the early PIR, the BrdU incorporation is higher in the cells compared to later periods, indirectly indicating the DSB repair. Hence, BrdU incorporation was measured under both unstabilized and stabilized G4 conditions using an anti-BrdU antibody. As shown in Fig. 6B, the BrdU signal was elevated at 1 h PIR samples for both NMM-treated (NMM, N+) and -untreated cells (no ligand, N−), suggesting increased ssDNA during the initial period of repair. At 4 h PIR, the mean fluorescence intensity was reduced in control cells (N− 4 h); however, in NMM-treated cells (N + 4 h), a significantly higher fluorescence signal was detected. An increased pool of newly synthesized, BrdU-containing ssDNA in NMM-treated cells at 4 h suggests that the process of conversion of ssDNA to dsDNA is slowed compared to control cells (Fig. 6B and C). The slow process of ssDNA to dsDNA conversion indicates a delay in DSB repair kinetics in cells that were treated with G4 ligand, which is also seen in PFGE experiments.

Cellular 8-oxo guanine (8-oxoG) is one of the major products of oxidative DNA damage (60). The levels of 8-oxoG following gamma radiation were checked to evaluate its effect on G4 formation. As shown in Fig. 6D, cells treated with NMM (NMM, N+) exhibited higher levels of 8-oxoG even after 1 h (N *+* 1 h) compared to control cells (N − 1 h) (Fig. 6E). While no or minimal levels of 8-oxoG were observed under unirradiated conditions (see Fig. S6 at https://barc.gov.in/publications/mBio01639-25R1/index.html). This suggests a possible delay in 8-oxoG repair due to arrested G4 dynamics in the presence of G4 ligand. Additionally, NMM- or TMPyP4-treated cells also showed higher G4 formation at 1 h PIR (Fig. 5B), suggesting that guanine oxidation does not hinder G4 formation in this bacterium. Collectively, these findings suggest that the stabilization of G4 structures slows repair of DSBs and oxidative DNA damage repair, thereby sensitizing the *D. radiodurans* cells to G4-binding drugs following gamma radiation.

### RecQ helicase is involved in the regulation of G4 structural changes and genome maintenance

**Fig 7:**
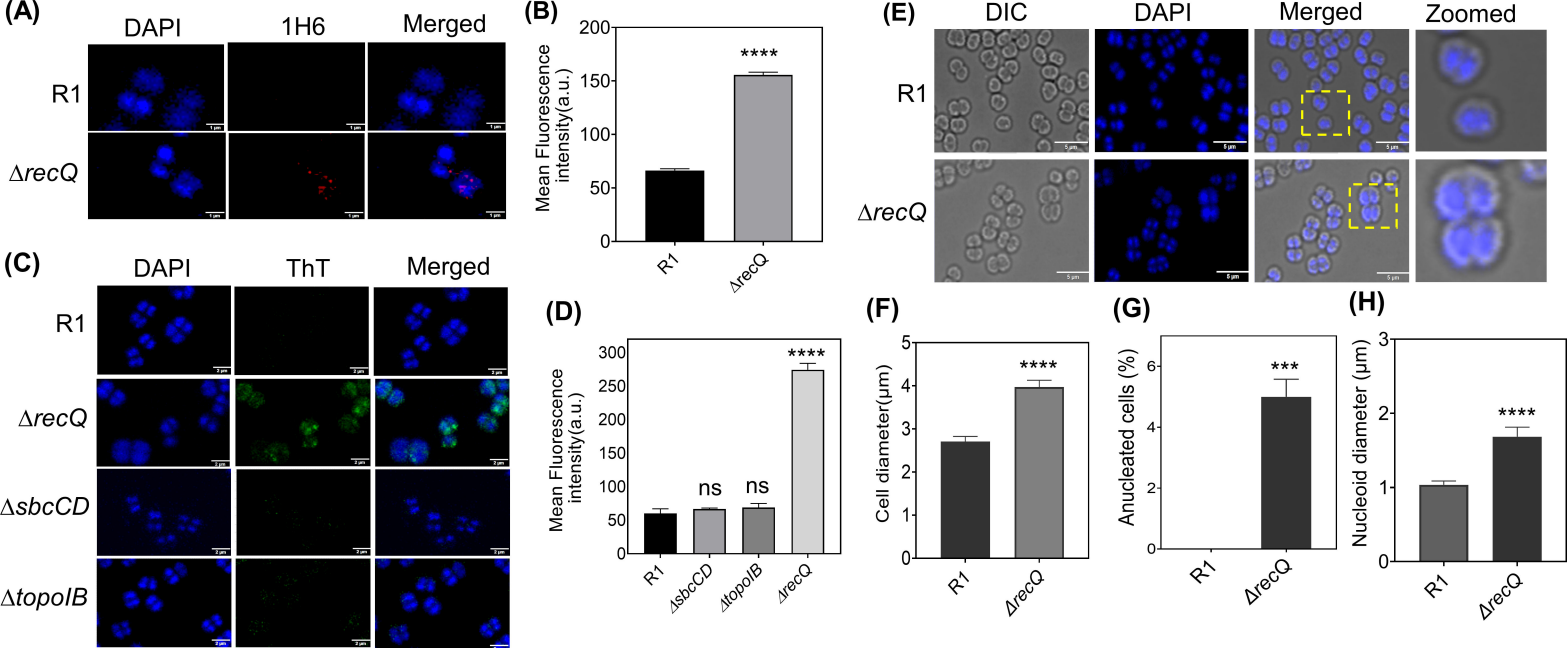
Involvement of RecQ helicase in the regulation of G4 structural dynamics and genome maintenance. (**A**) Immunofluorescence assay using an anti-G4 antibody (clone 1H6) of wild type (R1) and *recQ* mutant (Δ*recQ*). These cells were observed under DAPI (for nucleoid) and tetramethylrhodamine isothiocyanate (TRITC; 1H6) channels. The merged images represent the DAPI + TRITC channel. (**B**) The mean fluorescence intensity was depicted for R1 and Δ*recQ*. Data were analyzed using Student’s *t*-test (****P < 0.0001) and were plotted using GraphPad Prism software 8. (**C**) Fluorescence microscopic examination of wild type (R1), Δ*recQ,* Δ*topoIB,* and Δ*sbcCD* cells of *D. radiodurans*. The cells were grown and stained with DAPI and ThT. These cells were visualized under DAPI (for nucleoid) and FITC (ThT) channels. Each image is represented in separate as well as merged channels. The scale bar is 2 µm. (**D**) The mean fluorescence intensity was calculated for 150–200 cells. Statistical analysis used Student’s *t*-test, with ns for *P* > 0.05 and **** for *P* < 0.0001; error bar represents the standard error. (**E**) Morphological alterations in Δ*recQ* mutants compared to wild type (R1). Microscopic images show the morphological changes observed in Δ*recQ* mutants compared to wild type (R1) under a microscope. Differential interference contrast (DIC), DAPI, and merged channels are shown. The scale bar is 5 µm. (**F**) The cell size distribution, (**G**) the anucleated cell population, and (**H**) the nucleoid size distribution. The GraphPad Prism software 8 was used for statistical analysis of the different morphologies. The *P*-values attained at 95% confidence intervals are depicted as *** for <0.001 and **** for <0.0001. Each experiment was repeated thrice, and biological replicates were included.

The RecQ family of helicases in the eukaryotic system is known to resolve G4 DNA structures. Earlier studies have shown that in *D. radiodurans,* RecQ plays an important role in the metabolism of G4s (30). Hence, the *in vivo* presence of G4s in ∆*recQ* under normal conditions was examined by microscopy. The G4s were monitored with an anti-G4 antibody (clone 1H6). The results suggest that the formation of quadruplex structure was increased in ∆*recQ* under normal conditions compared to the wild type, R1 (Fig. 7A and B). Even ThT staining revealed that Δ*recQ* cells exhibited a higher intensity of ThT under normal conditions (Fig. 7C and D). Interestingly, these foci resemble those observed in wild-type cells at 1 h PIR (Fig. 4B). Thus, a greater abundance of G4 structures in the Δ*recQ* mutant was detected.

Earlier, it was reported that Mre11 in eukaryotes and topoisomerase IB in *D. radiodurans* are involved in the resolution of G4s (61, 62). Additionally, the Δ*topoIB* was shown to exhibit a higher proportion of anucleated cells than wild type (13). Hence, the formation of G4 was checked in Δ*topoIB* as well as in Δ*sbcCD* (SbcCD complex, a prokaryotic Mre11-Rad50 homolog) (63). The ThT intensity in Δ*topoIB* and Δ*sbcCD* cells was found to be comparable to that in wild-type cells (R1), suggesting similar levels of G4 structures (Fig. 7C and D). These results indicate that RecQ may serve as the major G4-resolving helicase in *D. radiodurans*. To explore this further, the ThT fluorescence intensity was checked in response to different stresses, such as gamma radiation, UV, and H_2_O_2_ treatments, in the *recQ* mutant. The ∆*recQ* cells showed similar ThT fluorescence intensity to the untreated control, suggesting increased levels of G4 structures in these cells under normal and all cellular stress conditions (see Fig. S7A at https://barc.gov.in/publications/mBio01639-25R1/index.html). Various cellular parameters such as cell size, nucleoid diameter, and the percent of anucleated cells in a population of ~150–200 cells were assessed as these parameters are interconnected and play a role in genome maintenance (Fig. 7E). The results revealed statistically significant changes in the average size of the cells (Fig. 7F), anucleated cell population (Fig. 7G), and diameter of the nucleoids (Fig. 7H) in Δ*recQ* compared to R1, indirectly suggesting G4’s role in maintaining genome stability. However, this needs to be investigated further. Taken together, these results support that RecQ helicase may play a central role in the regulation of G4 structure folding and unfolding dynamics in *D. radiodurans*.

## DISCUSSION

G4-forming sequences have been identified in bacterial genomes, where these sequences are non-randomly distributed and predominantly associated with gene regulatory regions. For instance, a study conducted on 89 pathogenic bacterial strains revealed that G4s exhibited uneven and non-random distribution. These are mostly found within pathogenicity islands, implying that stable quadruplex structures may influence processes related to pathogenicity (64). However, in contrast to eukaryotes, fewer studies probed the *in vivo* function of G4s in bacteria (65, 66). *D. radiodurans* contains a GC-rich genome. Earlier findings showed that G4s present in the promoter regions of most DNA repair genes regulate their expression during the PIR period (21). Here, the genome analysis revealed that certain deinococcal genes contain PQS at their 5′ region following the transcription start site or toward the 3′ end preceding the stop codon. A few PQS were also identified in intergenic regions. All the PQS studied here folded into different topologies depending on the presence of K^+^ or Na^+^ in the solution. Only one putative G4, identified in the intergenic region between *cpa* and *miaB,* showed stable G4 folding with Li^+^ ions. However, how the simultaneous presence of different monovalent cations will influence the G4 structure dynamics needs to be explored further.

In *D. radiodurans,* cellular accumulation of manganese (Mn^2+^) in the form of low molecular weight complexes plays a crucial role in protecting the proteome from oxidative damage and radiation (67). In this study, different concentrations of Mn^2+^ ranging from 0.1 mM to 10 mM could not support the *in vitro* formation of the examined deinococcal G4 structures. But 10 mM of Mg^2+^ supported the folding of G4 structures. Earlier, it has been reported that K^+^ (cationic radius 1.3 Å) is the best fit for occupation of the quadruplex helical core, whereas certain divalent transition metal cations like Mn^2+^, Co^2+^, and Ni^2+^ (cationic radius 0.6–0.8 Å) are a poorer fit within the quadruplex helical core (68). Hence, the possibility exists that the intracellular levels of Mn^2+^ could interfere with the G4 structures formed under normal growth conditions. This also aligns with the observations that cells are not sensitive to G4 ligands and have low *in vivo* G4 structures under normal growing conditions. Furthermore, even 0.1 mM Mn^2+^ along with 100 mM K^+^ failed to stabilize the G4 structures. But beyond its function as an antioxidant, the mechanism by which Mn^2+^ in low molecular weight complexes could modulate G4 structure dynamics remains unclear.

To explore the function of G4s in bacteria, many studies have used *in silico* analysis combined with *in vitro* or *in vivo* experiments using G4-binding ligands or dyes. Here, G4-specific antibodies and ThT were used to observe the G4 formation in *D. radiodurans*. Surprisingly, under normal growth conditions, G4s are barely detected, but following exposure to stress, especially to gamma radiation, the *in vivo* frequency of G4s was increased. Post-irradiation, DNA damage and the subsequent ssDNA generation during the dsDNA break repair pathway (ESDSA) might favor the G4 formation. It has been reported earlier that the levels of G4 in the cytoplasm increase under oxidative stress, modulating translation through stress granule formation (69). Similarly, stress also induces stable RNA quadruplex folding (70). The presence of G4s in different regions of the human genome leads to differential radio sensitivity (71, 72). In *D. radiodurans,* the G4s formed might aid in radioprotection, transcription, or DNA repair by recruiting proteins. The current observations, like differential effects of native G4s on endogenous gene expression, G4 structure dynamics during PIR, and delayed DNA repair upon G4 stabilization, support the G4 role in DNA damage repair in this bacterium.

Among all the bases, guanine is most susceptible to oxidation and readily forms 8-oxoguanine when exposed to reactive oxygen species. Studies have shown that 8-oxoG influences G4 folding and how DNA repair and transcription proteins recognize or modify it (73). *D. radiodurans* showed high levels of 8-oxoG shortly after gamma radiation exposure. The observed higher frequency of G4s at 1 h PIR suggests that 8-oxoG might not inhibit G4 formation in this bacterium. Previously, it has been reported that an additional G-track present near the core G4 tracks in the VEGF promoter can act as a “spare tire” This means that a damaged guanine base can be looped out, facilitating alternative G4 formation using the extra G-track. The looped-out damaged base may then either be repaired or increase the gene expression by recruiting transcription factors (74, 75). The possibility of similar mechanisms operating in this bacterium exists, as our earlier studies demonstrated that the promoters harboring putative G4 motifs of *recQ*, *mutL,* or *recA* increased the expression of a reporter gene post-gamma irradiation. Upon analysis, it was found that the promoters of *recA* and *recQ* contain an extra fifth G-track within 13 bp of their core G4-track (see Fig. S7B at https://barc.gov.in/publications/mBio01639-25R1/index.html). Additionally, considering the GC-rich deinococcal genome (~65% GC content), multiple putative G-tracks may act as spare tires. However, the possibility of G-track near the promoters acting as spare tires needs to be explored further.

RecQ helicases are broadly involved in DNA repair pathways, including homologous recombination. The helicases of the RecQ family, such as BLM, WRN, and *E. coli* RecQ, are involved in unwinding the G4s. The absence of some of these proteins, like BLM, and WRN, leads to genome instability in eukaryotes (76, 77). Earlier, studies from our group have also shown that in *D. radiodurans*, RecQ plays an important role in the resolution of G4s (30). In this study, the Δ*recQ* mutant displayed elevated G4 levels under normal growth conditions, comparable to wild type. In contrast, the loss of G4-resolving proteins like topoisomerase IB did not result in any increase in G4 levels under normal conditions. The Δ*recQ* mutant also showed a larger number of anucleated cells and altered nucleoid diameter. Since cellular and nucleoid morphology can also provide information about genome stability, all these results indirectly indicate genome instability in Δ*recQ*. This suggests that RecQ may be the primary G4-resolving helicase in this bacterium.

**Fig 8:**
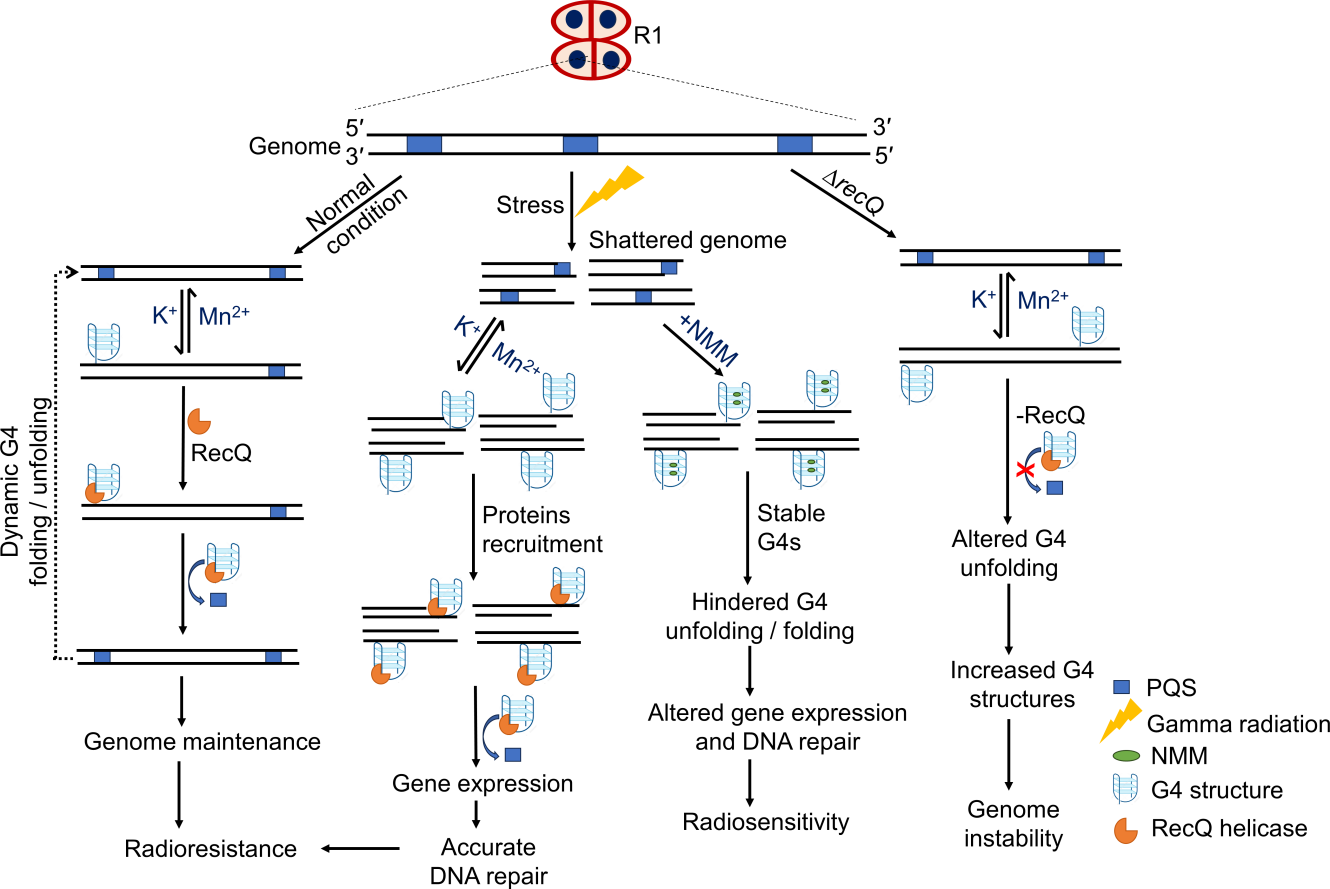
Schematic representation summarizing the *in vivo* G4s formation and its impact on gene expression and genome stability in *D. radiodurans*. Numerous PQS are located in the genome of *D. radiodurans*. Under normal growth conditions, intracellular G4 formation is either low or its dynamics are fast due to the presence of Mn^2+^ or proteins like RecQ. This enables the cells to maintain normal levels of gene expression and stable genome inheritance. When the bacterium is subjected to stress (γ-radiation), due to a shattered genome, the frequency of G4 formation increases, which may be involved in upregulation of DNA repair genes, recruitment of proteins, or radioprotection. However, when the G4 structure is stabilized (+NMM), the repair process gets delayed and altered gene expression, leading to a radiosensitive phenotype. The absence of RecQ helicase(-RecQ) increases the frequency of G4s, which may lead to genome instability. All these findings suggest that in *D. radiodurans*, the frequency of G4 formation is under tight regulation and the G4s are associated with the cellular processes involved in DNA damage repair and genome maintenance.

This study is the first to report *in vivo* evidence of G4 formation as well as the positional effect of native G4s on endogenous gene expression in a bacterium. As summarized in Fig. 8, numerous PQS are located on the GC-rich genome of *D. radiodurans*. Under normal growth conditions, detectable levels of G4s are not formed, possibly due to a higher intracellular concentration of Mn^2+^ or the activity of G4 helicases that may resolve the G4s. However, when exposed to stress, especially to gamma radiation, the G4 structures accumulate in cells. These G4s formed may regulate the transcription of genes or DNA repair by recruiting different proteins. Other factors, such as 8-oxoG levels and Mn^2+^ in low molecular weight complexes, may further influence the G4s formation or their role in recruiting proteins to specific genomic sites. The arrest of G4 structure folding or unfolding dynamics by G4 stabilizers like NMM may result in stable secondary structures that interfere with the DNA repair process, causing a radiosensitive phenotype. At the same time, accumulations of G4s in the absence of RecQ may impede the cellular processes leading to genome instability. Thus, during evolution, extremophiles like *D. radiodurans* may have utilized G4s to combat cellular stresses. Further studies on regulatory pathways or various proteins that maintain the G4 levels in this bacterium under different cellular conditions are crucial to understand the G4 biology in prokaryotes.
